# Supplementary material for: Patterns of Intron Gain and Loss in Fungi
Source: PLoS Biol. 2004 Nov 30;2(12):e422. doi: 10.1371/journal.pbio.0020422 (PMC532390; doi:10.1371/journal.pbio.0020422)
Supplement: Table S1 — Also available at http://genes.mit.edu/NielsenEtAl/. (4.3 MB ZIP). [file pbio.0020422.st001.zip › NielsenEtAl/html/703.html]

AN4245.1.NCU04721.1.MG07051.1.FG04738.1


```
 CLUSTAL W (1.82) Multiple Sequence Alignments - Introns Inserted


Sequence 1: MG07051.1	837 aa
Sequence 2: FG04738.1	748 aa
Sequence 3: NCU04721.1	780 aa
Sequence 4: AN4245.1	723 aa
Alignment Length: 873 aa
Number Identitical Residues: 245 aa
Alignment Score (without introns) 13511


MG07051.1 	MSERQVEAHRLLSEYPKTATPDVNSGLVTGTAIPRRNPRKTSPSVAFLSIVTAALLSLSF
NCU04721.1	-------------------MPYYRQDRGGGMAPPRRLITIAVSVVSFFILTALAIAQFGP
FG04738.1 	--------------------------------------MHTVTSSKFVHIL-LVYLTLGT
AN4245.1  	-------------------------------------MANFRLYLAFVGTFIFAVVLLQL
          	                                              *.     .   :  

MG07051.1 	LFITCSSPATWTWLASTLHPSLQHAETGGAETGNAGAAADRYLIGVGKADITG~PVVEIN
NCU04721.1	SSELHARKLAQRIEHAQRANPQSIPRDTPLG--------DKYLIGVGKADVTG~PVVEVG
FG04738.1 	FLVQLATCLAFNHLPKDTHNLLSKRADG-----------DVHYLGVGKADITG~PVVELN
AN4245.1  	ISVLHGFDDSPVLEASLQWHDRSSNYVGDD---------SIFVVGAGKADITG2PVVEVG
          	     .   :            .                . . :*.****:** ****:.

MG07051.1 	LMGYADPNQVGSGLRQR~LYSRAFMVGDLAHPADRFVYLVLDTQSGDTAVRYGIISALQS
NCU04721.1	LGGYADTSQVGSGLRQR~LYSRTFIIGETKNPKNRVVYIVLDTQSGDTAVRNGVLDALKG
FG04738.1 	LMGYAELSQTGTGLRQR~IYSRAFIVGNPDKPKERIVYVVLDAQSGDTAVRRGILEGIAA
AN4245.1  	FGGYADLDQVGTGLRQR2LYSRAFIVANPNNLEQTWIYIVLDTLTGDTGVRDGVLKGLAE
          	: ***: .*.*:***** :***:*::.:  :  :  :*:***: :***.** *::..:  

MG07051.1 	LGPEYAHYGHHNVAVTGTHSHSGPGGWLNYLLPQITSKGFDH~QGYRAI~VDGAVLSIRR
NCU04721.1	MGDEYSVYGQSNIALTGTHSHSGPGAWFNYLLPQITSLGFSK~QSYQAI0G---------
FG04738.1 	LGSDYAVYTSDNVAITGTHSHSGPAGWNNYLMPQISALGLNQ~QSYQAI~VDGAVLSIKR
AN4245.1  	LGSDYS-----------------------------------R2QSYNAI~VDGVLLSIRR
          	:* :*:                                   : *.*.**  ..   :   

MG07051.1 	AHESLTPGYLYVGKTKVAGANINRSLFAYLANPEVERNRYNVSSEDDGSVEKEMTLLKFQ
NCU04721.1	--------YLDIGTTDINDGNNNRSPSAYLANPESERAKY------PFNTDKTLTLLRFR
FG04738.1 	AHESLSKGRLSVGKTRIDDVNINRSLYAYQQNPASERQKY------KDEVDKELTMLMFT
AN4245.1  	AHESLAPGRLTFGTIDVEDANINRSPYSYDANPEEEKARY------PHNVDKTLELLRFD
          	: .: : . * .*.  : . * ***  :*  **  *: :*        ..:* : :* * 

MG07051.1 	RASDWRHLGILTWFPTHGTSMLGNNTLITGDNKGVAAYLFEKSMRYDVSAVPGVEFVAGF
NCU04721.1	RASDLKSVGVLTWYPVHGTSVFQNNTLVNADNKGVAAYLFEQSVKGDSRAADG--FVAGF
FG04738.1 	REEDNKVTGVLTWHSVHGTSLHMNNTLVAGDNKGVSAYLLEKAVRGTDGAAD--DFVAGF
AN4245.1  	RENDNKTMAVLTFFPVHGTSLYGNNTLASGDNKGVAAWLFERSVQDDSRFAN--DFVAGF
          	* .* :  .:**:...****:  ****  .*****:*:*:*::::     .   .*****

MG07051.1 	SQASVGDTSPNVKGAWCEDGSGLMCSFENSTCADGKAQACHARGPFFGRDDGGTASCFEI
NCU04721.1	SNANVGDTTPNTLGAYCDDGTGAACDFESSTCADGKVQACHGRGPLFQKLDKGVSSCYEI
FG04738.1 	SQAAVADTTPNVEGAWCEDGSGKQCDFKDSTCG-GKTETCHGRGPFWGLDDGGTKSCWEI
AN4245.1  	SQSNVGDTSPNILGAWCDDGSGEECRYSDSTCG-GQSTTCHGRGPFFREDSYGAKSCFEI
          	*:: *.**:**  **:*:**:*  * :..***. *:  :**.***::   . *. **:**

MG07051.1 	GMRQFKPARELY~DKLLLSEGTRISGGMVKSFHTFHDMSRFEFAHPRNSSQIIRTCPAAL
NCU04721.1	GRKQYAGAKNVW0TSLD-SSSTPVVGSTVRSFHYFHDMRYYKFNLD--NGTEVQTCPAAL
FG04738.1 	GRRVFDQAFKLY~DQMKDGSGDAVSGTSVVGYHSFHDMSDYSFILP--NGKTAKTCSAAF
AN4245.1  	GRRQYSAAKELY~SQMETNAIQIRKSSQVSSFHLFEDLKGYTFQSP-FNSSTLTTCSAAL
          	* : :  * :::  .:  .      .  * .:* *.*:  : *     ..    **.**:

MG07051.1 	GYSFAAGTSDGPGVFDFTQHDGDSSNTSPVWRAVSNMLKKPSDEQVACHAPKPILLDVGE
NCU04721.1	GHSFAAGTSDGPGAFDFTQGDSGAPNN-PFWSVVGGLLRVPSAEQAACQKPKPILLDVGE
FG04738.1 	GYSFAAGTTDGPGYFDFKQGDSGSPDASPLWAIVSKFLRNPTKEQVACQSPKPILIDAGE
AN4245.1  	GFSFAAGTTDWPGYFDFTQNDTTPAERNPLWYIARGFLHTPTPEQRKCQEPKDVLLDVGE
          	*.******:* ** ***.* *  ..: .*.*  .  :*: *: **  *: ** :*:*.**

MG07051.1 	VSRPYQWTPNIVDVQLFRVGQLAIIVSPGEATTMAGRRWKESVERAFVGFLDTERNDADG
NCU04721.1	MDNPYVWTPNIVDIQSFRVGQFFMIISPSEATTMSGRRWRDAVK-------------AAA
FG04738.1 	ITLPYAWAPNIVDIQMLRVGNFFIIVSASELTTMSSRRWRESIS-----------NEIQG
AN4245.1  	MSLPYAWTPNIVDIQLHRIGQLIIVTSTSEVTTMAGRRWREAIA---------------K
          	:  ** *:*****:*  *:*:: :: *..* ***:.***::::                 

MG07051.1 	NEDSDSEQLSPIVVLGGPANSYTHYITTEEEYGIQRYEGASTLYGPHTLDAYINVTLSHL
NCU04721.1	KQQKLTGSTEPVVVLGGPANTYAHYVATPEEYAVQRYEGASTLFGQYELPAYINLTLSAL
FG04738.1 	R-GSLGVDIKPIVVAGGPGNTYAHYVTTPEEYDAQRYEGASTVHGRHSLDAYINLTSTYV
AN4245.1  	SARDILSIFDPLVVLGSPANSYAHYVTTEEEYSRQRYEGASTLYGPNTLAAYVNLTLTYL
          	   .     .*:** *.*.*:*:**::* ***  ********:.*   * **:*:* : :

MG07051.1 	PYLFAGGKGSPGNGGSSPHHEDPSSFPPDNTNRSLSFITGVLRDGTKPFKSFGDVLTDVF
NCU04721.1	PYLSP-------SSTSSPPPG---PSPPDNRDDSLSFITGVVYDGAPSSKPFGTCITQPS
FG04738.1 	GYLLG------EYGAKKPPTG---PAAPDNRKNSLSLVTGVVYDNPKAGTKFGNIVKDVS
AN4245.1  	PYLDES-----AAAGQYPEPS--GIEPPINTEKSLSFIPSVVYDGHPIGKAYGDIITSAG
          	 **  .       . . *     .  .* * . ***::..*: *.    . :*  :..  

MG07051.1 	SDEEKKMPKLHFRRGVDIVRAVFVGANPRNNLRLEETYASVEKLDVARGSVWKTVRTDAD
NCU04721.1	---------SSYTRGSVVT-AVFQGANPRNNLRLEGTYAAVEKLG-ADGKTWTQVRSDED
FG04738.1 	--------KSKFKIGETIT-ATFVGANPRNNLHLESTYAAVEKKD---GSKWTQVRSDED
AN4245.1  	--------NTRYAPGDVAS-ATFIGANPRNNLRLESTFAAVERQT--DDGHWETVRTDSD
          	        .  :  *     *.* ********:** *:*:**:     .  *  **:* *

MG07051.1 	WTLVFRWRRVSSVLATSEAEISWETESWADP---------GVYRLRYFGDSKS-LGGDIK
NCU04721.1	WNMTYEWKRTNGLLGHSEVTIRWETRTESNGER---DDPTGTYRIRYYGDAKAAITGKIT
FG04738.1 	WDLVFEWKRLDGLLGSSEVQISWETG-WQDAK----DIKAGTYRLSYNGDSKAPLTGKIT
AN4245.1  	WSLVYRWKRTNTVLGHSEVTLQWEIEDDYYAVGSPRPVQAGVYRFHYYGDAKG-LNGRIE
          	* :.:.*:* . :*. **. : **         :.    :*.**: * **:*. : * * 

MG07051.1 	PFEGTS~SVFVLV--------------------------
NCU04721.1	EFQGVK1TRCIRPSHGKRSESPDQTQYWGKKETEIPTTC
FG04738.1 	GFTGKS~GEFEIA--------------------------
AN4245.1  	AFEGVD~EPFTVVV-------------------------
          	 * * .
```
